# Modeling Fragile X Syndrome in *Drosophila*

**DOI:** 10.3389/fnmol.2018.00124

**Published:** 2018-04-16

**Authors:** Małgorzata Drozd, Barbara Bardoni, Maria Capovilla

**Affiliations:** ^1^Université Côte d'Azur, CNRS, IPMC, Valbonne, France; ^2^CNRS LIA (Neogenex), Valbonne, France; ^3^Université Côte d'Azur, INSERM, CNRS, IPMC, Valbonne, France

**Keywords:** Fragile X Syndrome, FMR1, Fragile X Mental Retardation Protein, Drosophila, dFRM1, neuromuscular junction, mushroom bodies, behavior

## Abstract

Intellectual disability (ID) and autism are hallmarks of Fragile X Syndrome (FXS), a hereditary neurodevelopmental disorder. The gene responsible for FXS is Fragile X Mental Retardation gene 1 (*FMR1*) encoding the Fragile X Mental Retardation Protein (FMRP), an RNA-binding protein involved in RNA metabolism and modulating the expression level of many targets. Most cases of FXS are caused by silencing of *FMR1* due to CGG expansions in the 5′-UTR of the gene. Humans also carry the FXR1 and FXR2 paralogs of FMR1 while flies have only one *FMR1* gene, here called *dFMR1*, sharing the same level of sequence homology with all three human genes, but functionally most similar to *FMR1*. This enables a much easier approach for *FMR1* genetic studies. *Drosophila* has been widely used to investigate *FMR1* functions at genetic, cellular, and molecular levels since *dFMR1* mutants have many phenotypes in common with the wide spectrum of *FMR1* functions that underlay the disease. In this review, we present very recent *Drosophila* studies investigating FMRP functions at genetic, cellular, molecular, and electrophysiological levels in addition to research on pharmacological treatments in the fly model. These studies have the potential to aid the discovery of pharmacological therapies for FXS.

## Introduction

The Fragile X Syndrome (FXS), previously known as Martin-Bell syndrome or marker X syndrome or FRAXA, is the first X-linked intellectual disability (ID) syndrome described involving a DNA alteration and the most frequent heritable monogenic form of ID (reviewed in Penagarikano et al., 2007; Santoro et al., 2012; Hayward et al., 2017). Human FXS patients present severe ID often accompanied by an increase in Autism Spectrum Disorder (ASD) traits and other phenotypes like delayed development, hyperactivity, attention deficit, hypersensitivity to sensorial stimuli, anxiety, aggression, sleep, cardiac disorders, and epileptic seizures (reviewed in Hagerman, 2002; Garber et al., 2008; Utari et al., 2010; Santoro et al., 2012; Hagerman et al., 2014; Kidd et al., 2014; Maurin et al., 2014; Schaefer et al., 2015; Dahlhaus, 2018). These abnormalities can be explained by defects in neuronal development and maturation. Some patients also present characteristic morphological facial traits, macrocephaly, flat feet, and male macroorchidism. The first morphological phenotype observed in FXS patients was the presence of abnormalities in the spines (Comery et al., 1997; Irwin et al., 2001). More recently, ElectroEncephaloGraphy (EEG) and Magnetic Resonance Imaging (MRI) have evidenced volume and Event Related Potential (ERP) defects in FXS patients (Devitt et al., 2015).

FXS was initially associated with an X-chromosome fragile site (an isochromatid gap in metaphase chromosomes) in position Xq27.3 (Harrison et al., 1983). In 1991, this site was mapped to a CGG trinucleotide expansion in the 5′ non-coding region of a gene named *Fragile X Mental Retardation 1* (*FMR1*), the first gene associated with an X-linked ID (Verkerk et al., 1991). *FMR1* is 38 kb long and transcribed in a 4.4 kb full length mRNA that encodes a 632 aa protein called Fragile X Mental Retardation Protein (FMRP). Through alternative splicing, at least 12 different isoforms of 67–80 kD are produced. The CGG repeats are polymorphic in the population ranging from 5 to 54 repeats in normal individuals to more than 200 (full mutation) in severely affected patients (reviewed in Hayward et al., 2017). The repeat expansion results in hypermethylation of the CGG repeat, of a 5′ CpG island, and of flanking promoter sequences causing the reduction or absence of *FMR1* expression through an epigenetic mechanism involving *FMR1* mRNA (Colak et al., 2014). Several deletions and point mutations leading to the production of non-functional proteins have also been described (Okray et al., 2015 and references therein). Individuals with 55–200 CGG repetitions (premutation) do not present FXS symptoms, but may develop two other disorders: Fragile-X Primary Ovarian Insufficiency (FXPOI) (reviewed in Sherman et al., 2014) or Fragile X Associated Tremor/Ataxia Syndrome (FXTAS) (reviewed in Hall et al., 2016; Dahlhaus, 2018). FXTAS has been modeled in *Drosophila* by overexpressing 90 rCGG repeats alone fused to GFP, which causes a neuron-specific degeneration and the formation of inclusions (Jin et al., 2003; Qurashi et al., 2012).

In mammals, FMRP is nearly ubiquitous, present mainly in neurons (particularly in the cortex, hippocampus, and Purkinje cells) and in testes and absent from muscles and the heart (Devys et al., 1993). FMRP has two paralogs: Fragile X Related 1 (*FXR1*) and Fragile X Related 2 (*FXR2*). While FXR2 has a distribution comparable to that of FMRP, some isoforms of *FXR1* display a specific expression in brain while other isoforms are only present in muscle and heart (Khandjian et al., 1998; Bechara et al., 2007). These three proteins are members of the same family, namely the Related Fragile X Protein family, and are RNA-binding proteins mainly localized in the cytoplasm, although they carry a Nuclear Localization Signal (NLS) and a Nuclear Exportation Signal (NES) (Bardoni et al., 2000). Indeed, some isoforms of FMRP have also been localized in the nucleus (Eberhart et al., 1996; Bardoni et al., 1997). Collectively, these results have suggested that the three FXR proteins are able to shuttle between nucleus and cytoplasm to export their target mRNAs.

Three RNA-binding sequence motifs are the hallmarks of FMRP that may explain its function, *i.e*., two K homology (KH) domains and one Arginine-Glycine-Glycine (RGG) box. The main function of FMRP is to regulate translation and indeed it has been found associated with polyribosomes in different cell lines and, importantly, in the brain (Khandjian et al., 2004). Although FMRP mainly acts as a repressor, an activator function has been observed (reviewed in Maurin et al., 2014). Many methods have been used to identify FMRP targets (reviewed in Maurin et al., 2014; Davis and Broadie, 2017; Hayward et al., 2017): binding to biotinylated RNAs (Ashley et al., 1993), Cross-Link Immuno Precipitation (CLIP) (Darnell et al., 2011; Ascano et al., 2012; Anderson et al., 2016), Systematic Evolution of Ligands by Exponential Enrichment (SELEX) (Chen et al., 2003), yeast two-hybrid system (Ma et al., 2016), yeast three-hybrid system (Dolzhanskaya et al., 2003), and Antibody-Positioned RNA Amplification (APRA) (Miyashiro et al., 2003). Many of the identified targets have been involved in autism, other neuronal pathologies or gonadal development and many of them encode synaptic proteins (reviewed in Maurin et al., 2014). Finally, FMRP has been linked to the microRNA (miRNA) and Piwi-interacting RNA (piRNA) pathways in Mammals, *Drosophila*, and zebrafish (reviewed in Kelley et al., 2012; Specchia et al., 2017).

## Functional insights on FXS from *Drosophila* studies

The first model of FXS was the mammalian mouse model (The Dutch-Belgian Fragile X Consortium, 1994; Mientjes et al., 2006), which recapitulates some major patients' phenotypes (Dahlhaus, 2018 and references therein). However, ever since then, also research on *Drosophila melanogaster* has brought important knowledge on the basic mechanisms underlying FMRP function. The *Drosophila* homolog of *FMR1* was first identified in 2000 (Wan et al., 2000) and named *dfmr1*. Over the years, it has been called by many different names that are listed in the *Drosophila* database FlyBase (http://flybase.org/reports/FBrf0174476.html). It is now named *Fmr1* with a capital F, meaning that it has been identified through the human homolog *FMR1*. Here, we will call it *dFMR1* to distinguish it from the mouse gene (*Fmr1*). FlyBase names the protein Fmr1, but here we will call it dFMRP with the “d” indicating *Drosophila*. The *dFMR1* gene exhibits high sequence homology with all three human genes (FMRP, FXR1, and FXR2; Zhang et al., 2001; Coffee et al., 2010), but is most functionally related to *FMR1* (Coffee et al., 2010; see below). *dFMR1* is 8.7 kb long and transcribed in many different mRNAs of 2–4 kb encoding many different proteins of different sizes (http://flybase.org/reports/FBgn0028734.html). All functional domains are highly conserved with the two KH domains being 75% identical and 85% similar between *dFMR1* and *hFMR1* (Wan et al., 2000).

**Figure 1:**
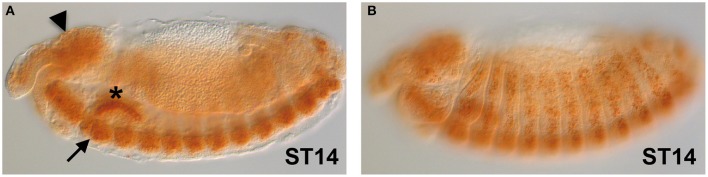
*dFMR1* expression in stage 14 *Drosophila* embryos**. (A)** Lateral view of a stage 14 embryo (middle focus) showing expression in the brain (arrowhead) and in the CNS (arrow). The salivary gland (asterisk) is non-specific background**. (B)** Lateral view of the same stage 14 embryo (surface focus) showing expression in several muscle precursors. The *dFMR1* anti-sense probe was synthesized from the full length EST-clone LD09557 (Drosophila Genomics Resource Center, Bloomington, IN, USA) linearized with EcoRI and transcribed with the T7 RNA polymerase using the Riboprobe Combination System kit (Promega, Madison, WI) and the DIG RNA Labeling Mix (Roche, Indianapolis, IN). *In situ* hybridization was performed as in Tevy et al. (2014) except that SA-HRP and TSA were diluted at 1:250. Images were acquired at the SPIBOC imaging platform of the Institut Sophia Agrobiotech (Sophia Antipolis, France) on an Axioplan II microscope using the ZEN software (Carl Zeiss, Germany).

The gene expression of *dFMR1* in embryos was explored soon after its cloning and observed in the Central Nervous System (CNS), in the somatic musculature, in pole cells, in the gut and in the gonads (Wan et al., 2000; Zhang et al., 2001; Schenck et al., 2002). In Figure 1, we show the expression of *dFMR1* at stage 14 by *in situ* hybridization with a full-length probe using the Tyramide Signal Amplification (TSA) (Tevy et al., 2014). High levels of expression are found in the brain (Figure 1A, arrowhead), in the CNS (Figure 1A, arrow) and in muscle precursors (Figure 1B), confirming the previously described pattern of *dFMR1* expression at this stage through a sensitive method.

In embryos, dFMRP has been localized in the brain, ventral nerve cord, and mesoderm (Zhang et al., 2001; Schenck et al., 2002; Dolzhanskaya et al., 2003; Ma et al., 2016), muscle attachment sites and dendritic tips of chordotonal organs, dendrites of the trachea-innervating neurons (Schenck et al., 2002), developing egg chambers (Zarnescu et al., 2005; Pepper et al., 2009), and in punctate cytoplasmic structures in cleavage-stage embryos in association with cytoplasmic RNP bodies (Monzo et al., 2006; Papoulas et al., 2010). In larvae, dFMRP has been detected in the CNS, the PNS, the eye disk, the testis, at low levels in muscles, in the Mushroom Bodies (MBs, the *Drosophila* learning and memory center; Schenck et al., 2002) and in dendritic arborization (DA) neurons (Lee et al., 2003). dFMRP is found exclusively in the cytoplasm of the soma of neurons (Zhang et al., 2001). Finally, in adults dFMRP has been found in the central brain and eyes (Zhang et al., 2001), in pupal and adult brain neurons (Morales et al., 2002), in specific cells of the adult brain (Morales et al., 2002), in antennal lobe projection neurons (Sudhakaran et al., 2014), and in the cell bodies of specific neurons of the MBs (Pan et al., 2004). Although the main tissue in which dFMRP is present is the CNS, these studies suggest that this protein also has non-neuronal functions, most of which still have to be dissected. Indeed, *dFMR1* has been involved by FlyBase (http://flybase.org/reports/FBgn0028734.html) in 58 biological processes, which are summarized in Table 1.

**Table 1:** Main phenotypes of loss of function *dFMR1* mutants.

**Phenotype**	**References**
**BEHAVIOR**	
Adult locomotion/Climbing	Zhang et al., 2001; Dockendorff et al., 2002; Inoue et al., 2002; Morales et al., 2002; Martinez et al., 2007; Banerjee et al., 2010; Adewoye et al., 2015; Novak et al., 2015; Monyak et al., 2016
Circadian rhythm	Dockendorff et al., 2002; Inoue et al., 2002; Morales et al., 2002; Banerjee et al., 2007, 2010; Gatto and Broadie, 2009; Siller and Broadie, 2011; Xu et al., 2012; Adewoye et al., 2015; Monyak et al., 2016
Courtship	Dockendorff et al., 2002; McBride et al., 2005; Banerjee et al., 2007, 2010; Chang et al., 2008; Choi et al., 2010; Tauber et al., 2011; Gross et al., 2015
Grooming	Tauber et al., 2011
Larval crawling	Xu et al., 2004; Coyne et al., 2015; Günther et al., 2016; Kashima et al., 2017
Olfactory learning and memory	Morales et al., 2002; Bolduc et al., 2008; Kanellopoulos et al., 2012; Andlauer et al., 2014; Sudhakaran et al., 2014; Choi et al., 2015; Monyak et al., 2016
Social behavior	Bolduc et al., 2010
Sleep	Bushey et al., 2009; van Alphen et al., 2013
Touch perception	Cvetkovska et al., 2013
**NEURAL PHYSIOLOGY/STRUCTURE**	
Bouton area	Pan et al., 2004; Gatto and Broadie, 2008; Reeve et al., 2008; Coffee et al., 2010; Friedman et al., 2013; Cavolo et al., 2016; Doll et al., 2017
Bouton/synapse number	Zhang et al., 2001; Zarnescu et al., 2005; Banerjee et al., 2007, 2010; Gatto and Broadie, 2008; Xu et al., 2008; Pepper et al., 2009; Coffee et al., 2010; Beerman and Jongens, 2011; Bhogal et al., 2011; Siller and Broadie, 2011; Friedman et al., 2013; Kashima et al., 2016, 2017; Mansilla et al., 2017
Calcium signaling	Tessier and Broadie, 2011; Gatto et al., 2014; Sudhakaran et al., 2014; Doll and Broadie, 2016
M B β-lobe crossing	Michel et al., 2004; McBride et al., 2005; Banerjee et al., 2007; Bolduc et al., 2008; Chang et al., 2008; Beerman and Jongens, 2011; Gross et al., 2015
Neural branching	Morales et al., 2002; Lee et al., 2003; Pan et al., 2004; Reeve et al., 2005, 2008; Gatto and Broadie, 2008; Tessier and Broadie, 2008; Xu et al., 2008; Cziko et al., 2009; Pepper et al., 2009; Coffee et al., 2010; Bhogal et al., 2011; Siller and Broadie, 2011; Friedman et al., 2013; Kim et al., 2013; Doll and Broadie, 2015; Myrick et al., 2015; Kashima et al., 2016
Neurite extension	Morales et al., 2002; Michel et al., 2004; Pan et al., 2004; Reeve et al., 2005; Gatto et al., 2014
Neural fasciculation	Schenck et al., 2003; Reeve et al., 2005
Neurotransrrission	Zhang et al., 2001; Martinez et al., 2007; Gatto and Broadie, 2008; Friedman et al., 2013; Doll et al., 2017; Franco et al., 2017
Synaptic growth	Zhang et al., 2001; Morales et al., 2002; Schenck et al., 2003; Abekhoukh et al., 2017; Doll et al., 2017
Branch/Neurite/NMJ/Synaptic length	Lee et al., 2003; Schenck et al., 2003; Reeve et al., 2008; Tessier and Broadie, 2008; Bhogal et al., 2011; Siller and Broadie, 2011; Cvetkovska et al., 2013; Kim et al., 2013; Gatto et al., 2014; Bozzetti et al., 2015; Myrick et al., 2015; Sterne et al., 2015; Abekhoukh et al., 2017; Doll et al., 2017; Kennedy and Broadie, 2017
Synapse structure	Zhang et al., 2001; Morales et al., 2002; Lee et al., 2003; Schenck et al., 2003; Michel et al., 2004; Pan et al., 2004; McBride et al., 2005; Reeve et al., 2005, 2008; Zarnescu et al., 2005; Banerjee et al., 2007, 2010; Bolduc et al., 2008; Chang et al., 2008; Gatto and Broadie, 2008, 2009; Tessier and Broadie, 2008; Xu et al., 2008; Cziko et al., 2009; Pepper et al., 2009; Coffee et al., 2010; Beerman and Jongens, 2011; Bhogal et al., 2011; Siller and Broadie, 2011; Cvetkovska et al., 2013; Friedman et al., 2013; Kim et al., 2013; Gatto et al., 2014; Bozzetti et al., 2015; Doll and Broadie, 2015; Gross et al., 2015; Myrick et al., 2015; Sterne et al., 2015; Cavolo et al., 2016; Kashima et al., 2016, 2017; Abekhoukh et al., 2017; Doll et al., 2017; Kennedy and Broadie, 2017; Mansilla et al., 2017
Synapse volume	Mansilla et al., 2017
**OTHERS**	
Adult eclosion	Dockendorff et al., 2002; Inoue et al., 2002; Morales et al., 2002
Aging	Martinez et al., 2007; Bushey et al., 2009
Apoptosis	Gatto and Broadie, 2011
Blastoderm cellularization	Deshpande et al., 2006; Monzo et al., 2006; Papoulas et al., 2010
Cell cycle	Deshpande et al., 2006; Monzo et al., 2006; Epstein et al., 2009; Callan et al., 2010, 2012; Papoulas et al., 2010
Germline development	Zhang et al., 2004, 2014; Costa et al., 2005; Deshpande et al., 2006; Megosh et al., 2006; Epstein et al., 2009; Yang et al., 2009; Bozzetti et al., 2015; Jiang et al., 2016
Heart rate	Novak et al., 2015
Oviposition	Kacsoh et al., 2015a,b; Jiang et al., 2016
Phagocytosis	O'Connor et al., 2017

It is interesting to note that the phenotypes of *dFMR1* mutants largely recall the pathological symptoms of FXS patients. For instance, some FXS patients have delayed motor development, which can be compared to uncoordinated behavior of flies measured by flight or climbing assays. Moreover, olfactory learning and courtship conditioning of Drosophila can serve to test learning and memory behaviors that are often impaired in FXS patients. Also the changes in neuron structure observed in FXS patients are mimicked in the fly model (reviewed in van Alphen and van Swinderen, 2013). The first *dFMR1* mutants were generated soon after its cloning (Zhang et al., 2001) and resulted viable and fertile as in humans. Nevertheless, mutant viability is highly sensitive to the genetic background, such that *dFMR1* mutants can become fully lethal in some backgrounds (see Morales et al., 2002) in a generation-dependent manner, a phenomenon that requires further study. These original alleles were loss-of-function excisions of hypomorphic EP insertions (producing over-expression of the gene in which they are inserted) presenting phenotypes upon over-expression in the eye (Zhang et al., 2001). Other null mutants were obtained by excision of other EP elements (Dockendorff et al., 2002; Inoue et al., 2002) or by EMS mutagenesis (Lee et al., 2003). Altogether, *dFMR1* mutants show defects in many different biological functions listed in Table 1. Over-expression studies in flies have also provided evidence for understating the mis-functions of mutant human FMRP as in the case of the assessment of a neomorphic function for a frameshift FMRP mutant (Okray et al., 2015). In summary, *dFMR1* mainly plays a crucial role in synaptic plasticity and this affects many neuronal processes that are important for fly behavior.

RNA-mediated interference (RNAi) enables the knockdown of a gene of interest at the post-transcriptional stage (Fire et al., 1998). Combining the RNAi with the UAS/GAL4 system (Brand and Perrimon, 1993) in flies makes it possible to down-regulate gene expression in certain tissues and/or at a desired stage of development. Using this combined mechanism, the promoter-GAL4 fusion drives the expression of the RNAi hairpin fragment under the control of UAS sequences (Piccin et al., 2001). Tissue- or stage-specific expression is achieved by the use of specific GAL4 drivers. For instance, *elav-GAL4* is usually used to trigger pan-neuronal expression, *mef2-GAL4* for pan-muscular expression and *Act5c-GAL4* for ubiquitous expression. Since 2007, the Vienna Drosophila Resource Center (VDRC; http://stockcenter.vdrc.at/control/main; Dietzl et al., 2007) has established many RNAi lines of *D. melanogaster*. These comprise around 12,671 (91%) of *Drosophila* protein-coding genes, making it the largest collection of RNAi lines for all model systems. Currently, there are three different types of UAS-RNAi stocks available: GD and KK with long hairpins and shRNA with short hairpin micro RNAs. The existence of such a broad array of RNAi lines provides many experimental benefits for biological studies, including those focusing on FMRP. For example, tissue-specific RNAi studies uncovered the role of dFMRP in early developmental stages of fly larvae. It was shown that dFMRP regulates glial-dependent proper timing of neuroblast reactivation during brain development (Callan et al., 2012). In another study, RNAi knockdown of *Torsin* revealed its involvement in locomotion. *Torsin* probably works together with *dFMR1* to regulate synaptic plasticity and *dFMR1* expression is altered in *Torsin* mutant flies (Nguyen et al., 2016). By creating the cardiac-specific *dFMR1* RNAi knockdown, *dFmr1* was also shown to be involved in regulating heart rate during development using the climbing assay. In this simple assay, adults are placed in a vial and tapped down. The time by which they reach the height of 5 cm is then measured (Novak et al., 2015). Some FMR1 patients present cardiac defects (Kidd et al., 2014) and changes in FMRP levels have been associated with structural and functional defects in zebrafish and mice (Mientjes et al., 2004; Van't Padje et al., 2009). On the contrary, in *Drosophila* no structural defects have been found, suggesting that *dFMR1* and *FMR1* regulate distinct targets. In a different study, hemocyte-specific *dFMR1* knockdown by RNAi causes a defect in immune cell phagocytosis of bacteria and increases *Drosophila* sensitivity to bacterial infections, suggesting that *dFMR1* is involved in the regulation of phagocytosis (O'Connor et al., 2017; reviewed in Logan, 2017). This study can provide further insights into the engagement of the immune system in FXS pathogenesis, especially considering the fact that some FXS patients exhibit defects in this system (O'Connor et al., 2017 and references therein). Aberrations in calcium homeostasis are connected with changes in neuron structure that probably cause the learning and memory deficits seen in FXS patients (Tessier and Broadie, 2011 and references therein). This was supported by RNAi knockdown of *dFMR1* during a critical period of development, which proved the importance of the dFMRP role in regulating calcium signaling in the learning and memory circuitry (Doll and Broadie, 2016). These studies highlight the value of the *Drosophila* system in detailed phenotypic analyses of FMRP function.

NeuroMuscular Junctions (NMJs) of *Drosophila* are simple synapses that resemble those present in the Vertebrate CNS. Thus, they are a good model for the study of synapses (reviewed in Menon et al., 2013). The neuromuscular system of *Drosophila* contains 32 motor neurons in each abdominal hemisegment. NMJ synapses show developmental and functional plasticity. They are large and individually specified, enabling their visualization. NMJs are composed of branches and three types of boutons (I, II, and III) that are oval structures hosting synapses differing in size, shape, physiology, and in the amount of sub-synaptic reticulum surrounding them. Type I boutons are glutamatergic and have been the focus of FXS studies. Immunohistological staining can be used to visualize these structures and observe different NMJ phenotypes. *dFMR1* is expressed pre-synaptically in motor neurons (Zhang et al., 2001), but post-synaptically in muscles (Zhang et al., 2001; Schenck et al., 2002). In *dFMR1* mutants, NMJs display increased synapse arborization and branching, increased synaptic bouton numbers, and elevated neurotransmission, whereas larvae over-expressing *dFMR1* show the opposite phenotypes (Zhang et al., 2001). These phenotypes recall the dendritic spine over-growth observed in mammalian mutants and in FXS patients (Irwin et al., 2001).

In addition to defects in NMJ synaptic architecture in neurons, *dFMR1* mutants also exhibit fecundity and testes dysfunctions, which can be used to evaluate non-neuronal requirements (Zhang et al., 2004). Coffee et al. examined the evolutionary conservation of *FMR1* and its paralogs in the *Drosophila* FXS model at the neuronal and non-neuronal levels (Coffee et al., 2010). In this study, out of the three human genes, only *FMR1* turned out to be able to restore the normal number of boutons in *dFMR1* null mutants. On the other hand, all three homologs rescued the sterility and testicular phenotypes. These results indicate that in neurons *FMR1* has a unique evolutionarily conserved role. In contrast, in non-neuronal tissues *FMR1, hFXR1*, and *hFXR2* are able to substitute each other (Coffee et al., 2010).

The larval NMJ has proved a powerful system to study genetic interactions occurring in the actin remodeling pathway that is altered in mammalian FMRP-null neurons (Castets et al., 2005; Pyronneau et al., 2017). FMRP has been shown to interact with Cytoplasmic FMRP Interacting Protein 1 (CYFIP1) *in vitro* (Schenck et al., 2001, 2003) and also in both mammals (Schenck et al., 2001) and *Drosophila* (Schenck et al., 2003; Abekhoukh et al., 2017). CYFIP1 is part of the WAVE regulatory complex (WRC) along with five other proteins involved in actin polymerization (reviewed in Cory and Ridley, 2002; Takenawa and Suetsugu, 2007). Human CYFIP1 has been linked to neurodevelopmental disorders such as ID, autism, schizophrenia, epilepsy, and Burnside-Butler (15q11.2 BP1-BP2 micro-deletion) syndrome (Madrigal et al., 2012; Waltes et al., 2014; Huang, 2015; Wang et al., 2015). Abekhoukh et al. (2017) utilized the fine genetic tools of *Drosophila* to investigate the genetic interactions between dFMRP and dCYFIP1. Through loss- and gain-of-functions studies, the authors showed that *dFMR1* and d*CYFIP1* have opposing functions on larval NMJ length: dFMRP represses while dCYFIP1 promotes synaptic growth at the NMJ in gene dosage studies using the presynaptic *elav-Gal4* driver. A rescue of the reciprocal NMJ length phenotypes is observed in double homozygous mutant animals. It should be noted that double homozygous mice are lethal, thus preventing a similar epistatic genetic analysis in the mouse model. Here, the advantage of *Drosophila* for synaptic plasticity and actin studies is that specific parameters can easily be monitored. Since *dFMR1* and *CYFIP1* are candidates for ID and autism, these studies on the fly pave the way to deeper and more refined studies in mice and in humans.

The first *Drosophila* target of dFMRP was the gene *futsch*, identified by RNA immunoprecipitation (Zhang et al., 2001). This gene encodes a microtubule-associated protein with homology to mammalian MAP1B. *futsch* and *dFMR1* have opposite phenotypes (undergrowth and overgrowth of synaptic boutons, respectively) and dFMRP binds *futsch* mRNA, negatively regulating its translation (Zhang et al., 2001). Binding of dFMRP was also found for the mRNA of the actin monomer binding protein *profilin* (encoded by *chickadee*; Reeve et al., 2005) and of the small GTPase Rac1 (Lee et al., 2003), whose loss- and gain-of-functions also have opposite phenotypes to those of *dFMRP* that are rescued by over-expression of both genes. dFMRP also binds the mRNAs of BMPR2 (Kashima et al., 2016), DSCAM (Cvetkovska et al., 2013), and the Ca^2+^/calmodulin-dependent protein kinase II (CaMKII) mRNA together with Ataxin-2 (Sudhakaran et al., 2014), confirming the involvement of *dFMR1* in the Ca^2+^ pathway. Nevertheless, there is still a need for high throughput studies to identify novel dFMRP targets.

MBs consist of bilateral clusters of ~2,500 neurons in the fly brain. In MBs, there are three types of intrinsic neurons: αβ, α′β′, and γ (Crittenden et al., 1998). α′β′ are a prerequisite for gaining olfactory memory, whereas αβ are required to retrieve memory (Krashes et al., 2007). MBs play a major role in olfactory learning and memory in *Drosophila*. Odor and courtship-based tests are frequently used to assess memory dysfunctions in this system (reviewed in Weisz et al., 2015). MBs are also involved in visual context generalization, information processing, locomotion, sleep, courtship conditioning, and choice behavior. The MB axons in *dFMR1* null mutants show architectural defects such as an increase in volume and branching, as well as abnormalities in synapse formation (Pan et al., 2004; Chang et al., 2008). *dFMR1* null mutants show morphological MB defects in the lobes, the most frequent of which is the failure of β-lobe arrest at the brain midline (Michel et al., 2004 and see Table 1), whereas over-expression of *dFMR1* causes the opposite phenotype (Reeve et al., 2005). These MB malformations can be restored through pharmacological treatment, but they are not indispensable for restoring memory (McBride et al., 2005). It has been shown that *dFMR1* expression requirements differ between lobe types in MBs. Simultaneous expression of *dFMR1* in α, β, and γ lobes is essential for learning skills. The lack of *dFMR1* expression in α and β lobes is sufficient to impair associative olfactory learning and memory, whereas the knock-down of *dFMR1* only in γ lobes does not exhibit detrimental effects on learning (Kanellopoulos et al., 2012). The MBs have also been the focus of calcium-signaling studies using a transgenic GCaMP calcium sensor (Doll and Broadie, 2016 and references therein). The MB neurons have been shown to be involved in activity-dependent processes during critical period development thanks to their ability to respond when illuminated by a blue light (see below; reviewed in Doll and Broadie, 2014). All the *dFMR1* phenotypes in the MBs correlate with learning and memory dysfunctions and make the FXS *Drosophila* model very appealing because of its easy *in vivo* analysis and the wide range of tools that have been developed (Ugur et al., 2016; Chow and Reiter, 2017).

Electrophysiological techniques have been used in *Drosophila* to study the effect of *dFMR1* loss-of-function and over-expression on synaptic transmission. Two-Electrode Voltage Clamp (TEVC) studies at NMJs in *Drosophila* showed that FMRP has clear pre-synaptic functions (Zhang et al., 2001), although photoreceptor synaptic transmission is normal in the mutants (Morales et al., 2002). Through TEVC recordings, Gatto and Broadie (2008) showed that presynaptic *dFMR1* expression in *dFMR1* mutants rescues the defects in NMJ structure, but not in neurotransmission, suggesting that dFMRP also has post-synaptic functions. On the other hand, in mice, FMRP was first shown to act post-synaptically (Huber et al., 2002) and only recently has also been found to play a pre-synaptic role in signal transmission (Koga et al., 2015; Myrick et al., 2015; Zhang et al., 2015) as in the fly. The possibility to target gene expression to specific cells through the UAS/GAL4 system (Brand and Perrimon, 1993), the wide availability of genetic mutants and the precise spatial and temporal resolutions make electrophysiology a very informative technique for FXS modeling in flies, and a useful complement to the advanced imaging studies described below.

Optogenetics is an *in vivo* technique that uses light to measure neuronal activity in living tissues and has also been exploited in the FXS *Drosophila* model. For example, dFMRP has been shown to play a cell-specific role in the regulation of activity-dependent calcium transients that is restricted to the early critical period (Tessier and Broadie, 2011; Doll and Broadie, 2016). Using optogenetic stimulation, it was recently established that *dFMR1* mutants show increased circuit excitability (probably due to reduced GABAergic lateral inhibition; Franco et al., 2017) and that dFMRP is required for the activity-dependent regulation of synaptic connectivity (Doll et al., 2017). These data may explain the deficits in olfactory behaviors and the hyper-excitation found in FXS patients. *Drosophila* has been used to study even sleep patterns in *dFMR1* mutants by electrophysiology and optogenetics. *dFMR1* mutants show deeper night-like sleep during the day (van Alphen et al., 2013), which is likely because of the FMRP function in synaptic remodeling. It is clear that, through *Drosophila*, the cellular and physiological processes involved in FXS pathology can be studied at a deeper level than in any other model system. In addition, flies have been used for pharmacological studies on FXS through their complex behaviors like for example olfactory learning and memory, courtship, circadian rhythm, crawling and sleep (see Table 1 and below).

## FXS in other model systems

*Drosophila* is not the only animal model used to study FXS. Here, we report some examples of other animal models that can help in FXS studies. Mammalian mouse and rat models have been predominantly used for FXS studies. Two different mouse (The Dutch-Belgian Fragile X Consortium, 1994; Mientjes et al., 2006) and rat (Hamilton et al., 2014; Tian et al., 2017) KO models have been generated. It should be noted that, in mice, the full CGG expansion does not cause methylation and *Fmr1* silencing (Brouwer et al., 2007) as it does in humans. Thus, all FXS studies in mice have been carried out using the KO animal (The Dutch-Belgian Fragile X Consortium, 1994; Mientjes et al., 2006). In general, the *Fmr1*-KO mouse is considered to be a good model for this disorder since it recapitulates most FXS phenotypes (deficits in learning and memory, hyperactivity, altered volumes of some brain regions, altered morphology of dendritic spines, and increased size of testis) and because it allows genetic experimentation. Also KO mutant rats present behavioral abnormalities related to ASD-like altered patterns of social interaction (Tian et al., 2017) and social play behavior (Hamilton et al., 2014), defects in visual attention (Berzhanskaya et al., 2016), and speech and auditory dysfunctions (Engineer et al., 2014). Rats have provided an excellent model for neuroscience and pharmacology as they have bigger brains than mice, are easier to train, can learn sophisticated behaviors and have an elaborated social repertoire; however, they are more expensive and much less genetically amenable than mice and flies. These recent studies on *Fmr1* KO mice and rats show that both of these models are useful to study the complex phenotypes of FXS patients. Nevertheless, although Vertebrate model organisms provide more precise insights into the human disease pathogenesis, they are much more difficult than flies to maintain, more time-consuming and incur considerable expenses. In addition, performing experiments on Vertebrate models is much more restrictive in the context of animal laws and often triggers ethical issues.

Other animal models have been generated to understand the physiopathology of FXS, such as the zebrafish, a small fresh water fish endogenous to South-East Asia, the frog *Xenopus laevis* and the marine mollusk *Aplysia californica*. Although zebrafish *fmr1* mutants generated by morpholino knock-down showed gross morphological defects in neurons (Tucker et al., 2006), true genetic null alleles later obtained by random mutagenesis resulted viable, fertile, and of normal morphology (den Broeder et al., 2009) likely due to off-targeting effects of the morpholino technology. Importantly, mutant zebrafish exhibit hyperactivity, learning deficits, impaired anxiety, and increased social behaviors like shoaling (Ng et al., 2013; Kim et al., 2014; Wu et al., 2017) maybe because of hyperactivity as in mice (Sorensen et al., 2015). The frog *fmr1* mutant was also obtained by morpholino KO and showed behavioral (Truszkowski et al., 2016) and FMRP level-sensitive neuronal defects (Faulkner et al., 2015). Finally, in *Aplysia*, basic neurobiological studies have evidenced the pre- and post-synaptic control of plasticity regulating long-term memory and a functional interaction with the Na^+^-activated K^+^ channel (K_Na_) Slack (reviewed in Abrams, 2012). Overall, these simpler systems have also facilitated further insights into the mechanisms of FXS pathology.

In comparison to vertebrate models, the more complex human behaviors do not always correspond to those of flies and many neurological diseases can only be modeled in certain aspects. This is mainly due to the fact that *Drosophila* and humans differ in anatomy and despite of having many orthologs in common their pathways exhibit many differences. In addition, although drug administration is much simpler in *Drosophila*, the potential toxicity is much tougher to predict in humans because of significant metabolic differences and complexities (Pandey and Nichols, 2011).

## Successful pharmacological treatments of FXS phenotypes in *Drosophila*

Since the *Drosophila dFMR1* phenotypes recapitulate the patients' symptoms, this model has been used to develop pharmacological treatments for the disease. One reason is that drugs can easily be administered in the standard fly food in which larvae feed and grow. This food is composed of cornmeal, agar and yeast and requires boiling. Recently, the Formula 4–24 (Carolina Biological Supply Company) medium has been exploited because it does not require heating and can simply be dissolved in room temperature water so that even heat-sensitive drugs can be tested (Kashima et al., 2017). Simple feeding has been used in the articles reported below, but many other methods to feed embryos, larvae or adults have been developed (reviewed in Pandey and Nichols, 2011). Another reason is that *Drosophila* does not carry vessels, but all of its organs bathe in hemolymph, which circulates thanks to a tubular heart in an open circulatory system. The fly blood/brain barrier is only made of a thin layer of glial cells presenting septate and gap junctions contrary to the Vertebrate one that is composed of glial cells and of endothelial cells forming tight junctions. Thus, the fly blood/brain barrier is simpler and allows for a better pharmacokinetic penetration (Pinsonneault et al., 2011; Limmer et al., 2014). Here, we discuss some examples of drugs successfully tested on flies to cure *dFMR1* phenotypes that affect the different pathways listed below.

Glutamate is the main excitatory neurotransmitter in the CNS. Metabotropic glutamate receptors (mGluRs) at synapses control Long Term Depression (LTD), which mediates synaptic plasticity, thus weakening the synaptic response to stimuli. mGluR-dependent LTD was initially found to be enhanced in *FMR1* mutant mice (Huber et al., 2002) and later in *Drosophila* (McBride et al., 2005). After the establishment of the “mGluR theory” of FXS by Bear et al. (2004), it was in *Drosophila* that inhibition of mGluR signaling was first shown to alleviate the behavioral fly phenotypes mimicking human FXS symptoms (McBride et al., 2005). In addition, a rescue of the defects in the fibers crossing the β-lobe of the MBs was observed in the treated *dFMR1* mutants. Interestingly, adult therapy is sufficient to restore normal courtship behavior and short-term memory, but not β-lobe crossing, suggesting that other morphological defects are responsible for the memory defects (McBride et al., 2005). Since then, much research has been carried out to pharmacologically correct this defect both in mice (reviewed in Hagerman et al., 2014) and in flies (Choi et al., 2010; Kanellopoulos et al., 2012). *dFMR1* mutants have less vigorous courtship behavior, learn normally, but forget. Treatments of larvae and adults either with the non-competitive mGluR antagonist 2-methyl-6-(phenylethynyl)pyridine (MPEP), three competitive mGluR antagonists or LiCl rescue naive courtship behavior, immediate recall memory and short-term memory of *dFMR1* mutants (Choi et al., 2010). Subsequently, Choi et al. (2010) compared naive courtship, locomotion, olfactory capabilities, learning, and memory between 20 day-old flies and 5 day-old flies. They found that the inhibitors of the Glutamate receptor pathway and Lithium rescue these phenotypes of *dFMR1* mutants to different degrees. The best rescue is obtained when the treatment is applied at both larval and adult stages. Learning is defective only in old flies, without the involvement of cell death. Treatment with four different mGluR inhibitors or LiCl exclusively during development rescues the learning, but not the courtship defects. Despite these successful treatments in pre-clinical trials, all clinical trials carried out so far have failed, although others are currently underway (https://clinicaltrials.gov). One reason could be the timing of the treatment or the need to combine different drugs since many other molecular mechanisms have been evoked as causative aspects of this disease. Importantly, in addition to FXS, many other neurodevelopmental disorders are correlated with defects in mGluR signaling.

As opposed to glutamate, GABA is the main inhibitory neurotransmitter in the brain. It functions through Gamma-AminoButyric Acid (GABA)_A_ and GABA_B_ receptors (R) that are fast-acting inotropic and metabotropic receptors, respectively. A decrease in GABA_A_ R expression and defects in GABAergic signaling were found in the *FMR1* Knock Out (KO) mouse (Idrissi et al., 2005; D'Hulst et al., 2006; Gantois et al., 2006; Sabanov et al., 2017). GABA_B_ R has been linked to mGluR signaling and its reduction associated with autism (reviewed in Hagerman et al., 2014). Many clinical treatments targeted to the GABAergic pathway have been attempted or are underway (reviewed in Braat and Kooy, 2015). Altered GABAergic circuitry, like depressed glutamic acid decarboxylase (GAD) levels, was also found in the *Drosophila* FXS model, particularly in neurons expressing *dFMR1* (D'Hulst et al., 2006; Gatto et al., 2014). In this system, through a fine mapping method of Mosaic Analysis with a Repressible Cell Marker (MARCM), Gatto et al. (2014) showed that *dFMR1* is not required for GABAergic neuron survival, but it regulates the architecture of GABergic neurons innervating the MBs. In addition, *dFMR1* GABAergic neurons display elevated calcium signaling (Gatto et al., 2014). To further underline the critical role of the GABA pathway in defining the FXR phenotype in the fly, it is worth mentioning that the down regulation of the GABA receptors in projection neurons of the antennal lobe observed in *dFMR1* mutants is sufficient to produce olfactory behavioral defects (Franco et al., 2017).

Thanks to the toxicity of glutamate for the *dFMR1* mutant, Chang et al. (2008) were able to carry out an unbiased screen for small molecules that can rescue the lethality of glutamate-treated *dFMR1* mutants, using the mGluR5 non-competitive antagonist MPEP as a positive control. The active compounds belong to biochemical pathways not targeting mGluR signaling, namely the GABAergic, the muscarinic, the serotonin, and hormone-related pathways. Notably, GABA and MPEP treatments were also able to rescue the β-lobe crossing in the MBs and the courtship behavior phenotypes. Indeed, a decrease in GABA_A_ R expression and defects in GABAergic signaling were found in the *FMR1* KO mouse (Sabanov et al., 2017 and references therein) and in the *Drosophila* FXS model, particularly in neurons expressing *dFMR1* (Gatto et al., 2014). This is the first chemical screen for FXS in flies and was made possible by the easily scorable phenotype of lethality, which revealed novel pathways implicated in FXS (Chang et al., 2008). GABA_B_ R has been linked to mGluR signaling and its reduction associated with autism (reviewed in Hagerman et al., 2014). Many clinical treatments targeting the GABAergic pathway have been attempted or are underway (reviewed in Braat and Kooy, 2015).

Another pathway that has been shown to be involved in FXS alterations is the Bone Morphogenetic Protein (BMP) signal transduction pathway (Kashima et al., 2016), controlling a number of developmental processes including nervous system development (Liu and Niswander, 2005). This pathway has been linked to anxiety and object exploration in mice (McBrayer et al., 2015). It was previously established that the LIM domain kinase 1 (LIMK1) co-localizes with the BMP type II receptor (BMPR2) in the neuronal terminals (Lee-Hoeflich et al., 2004). These two proteins bind to each other leading to activation of the LIMK1 catalytic activity. Once activated by BMPR2, LIMK1 promotes neural growth and dendritogenesis through phosphorylation and inhibition of cofilin (Meng et al., 2002; Lee-Hoeflich et al., 2004). The C-terminal domain (CTD) of BMPR2 plays a role as a repressor of BMPR2 translation and as an activator of LIMK1 function. Kashima et al. (2016) found that FMRP binds to the CTD of the BMPR2 and inhibits the translation of full-length BMPR2 in humans and mice. These data have been genetically confirmed in the *Drosophila* model. In the NMJs of *dFMR1* mutants, synaptic boutons are over-grown, whereas they are under-grown in mutants for the BMPR2 homolog *wishful thinking* (*wit*) (see also Eaton and Davis, 2005). Double *wit, dFMR1* mutants show a number of boutons as in single *wit* mutants, indicating that *wit* is negatively regulated by *dFMR1*. These results obtained in the fly encouraged the authors to carry out pharmacological studies in mice that revealed the *Fmr1* rescue potential of a LIMK1 inhibitor (Kashima et al., 2016). These cellular phenotypes correlate with the crawling behavior of *Drosophila* larvae, which has been used as a simple genetic and pharmacological screening tool for FXS treatment (Kashima et al., 2017). *dFMR1* mutant larvae crawl faster than wild-type larvae: heterozygotes for three different *dFMR1* alleles show increased larval locomotor activity correlated with an increased bouton number at the NMJ of muscle 6/7 in A3. This phenotype is rescued by a loss-of-function allele of *wit* in heterozygosis suggesting that it is due to an up-regulation of the BMPR2 homolog. The oral treatment with a pharmacological inhibitor of LIMK1 (downstream target of BMPR2, see above) restores the number of boutons in *dFMR1* mutants. Using a newly developed larval crawling assay and a sophisticated algorithm (LarvaTrack) for drug screening, the locomotion defects (distance and velocity) of *dFMR1* larvae were used as a readout of the larval bouton number phenotype (Kashima et al., 2017). In this case, LIMK1 inhibitors and puromycin rescue the *dFMR1* locomotion phenotypes in correlation with the bouton number. Hyperactivity and anxiety of *Fmr1-KO* mice are also ameliorated by treatment with a LIMK inhibitor, showing that this specific and simple assay developed in flies has considerable potential in the assessment of new drug therapies. Indeed, all of these results can be applied to the human condition because an increase in BMPR2 is observed in the prefrontal cortexes of FXS patients (Kashima et al., 2016), paving the way to drug trials in humans.

Neuronal Calcium Sensor 1 (NCS-1) and its *Drosophila* homolog *Frequenin* (*Frq*) 2 are Ca^2+^-binding proteins that play a role in the control of the synapse number: loss-of-function mutations increase the synapse number and over-expression decreases it. Human NCS-1 is involved in schizophrenia (Koh et al., 2003; Torres et al., 2009) and autism (Piton et al., 2008). Frq2 physically interacts with the guanine nucleotide-exchange factor *Ric8a* and the structure of their interaction has been resolved (Romero-Pozuelo et al., 2014). The *Ric8a* protein is localized at larval motor neuron terminals and is particularly abundant in boutons. *Ric8a* knockdown causes a reduction in the number of synapses, thus producing a phenotype opposite to that of Freq2 with Frq2 acting as a negative regulator of Ric8a in the control of the synapse number (Romero-Pozuelo et al., 2014). On the other hand, as in humans and mice (Irwin et al., 2001; Hutsler and Zhang, 2010), loss of *dFMR1* causes an increase in the synapse number and neuron volume (see Table 1). Mansilla et al. (2017) identified the aminophenothiazine derivative FD44 as a potent inhibitor of the *Drosophila* NCS-1/Ric8a interaction and showed that treatment of *dFMR1* mutant adults and larvae with this drug reduces their synapse number and volume at the glutamatergic NMJ of larval muscle fibers 6/7 of A3. In addition, FD44 restores normal associative learning. This indicates that, in the *dFMR1* mutant, the interaction between NCS-1 and Ric8a is unbalanced, probably because dFMRP controls *Frq2* transcription (Tessier and Broadie, 2011). Consistently, over-expression of both *NCS-*1 and *Ric8a* restores a normal number of synapses (Romero-Pozuelo et al., 2014). FD44 is an interesting drug because it is small and able to cross the blood/brain barrier and because the structure of the interaction with its target proteins has been clearly illustrated (Mansilla et al., 2017).

The Down Syndrome Cell Adhesion Molecule (DSCAM) is a conserved protein the levels of which have been found to be elevated in many brain disorders including FXS. This has been confirmed in the mouse and *Drosophila* models where it has been shown that FMRP binds the Dscam mRNA and down-regulates its translation (Darnell et al., 2001; Cvetkovska et al., 2013; Kim et al., 2013). *Dscam* encodes a transmembrane protein that is a member of the immunoglobulin superfamily of cell adhesion molecules involved in self-avoidance, synaptic target selection, and axon guidance. In pre-synaptic terminals, *Dscam* has been shown to interact with *Abelson tyrosine kinase* (*Abl*), which mediates the exaggerated presynaptic terminal growth followed by *Dscam* over-expression probably because it is activated by DSCAM (Sterne et al., 2015). As multiple FDA-approved Abl inhibitors were available, Sterne et al. (2015) tested whether ABL inhibition could restore normal presynaptic terminal growth in larvae over-expressing *Dscam* or mutant for *dFMR1*. The nilotinib inhibitor was found to have this property, which suggests that ABL is as a promising therapeutic target for FXS.

Insulin and insulin-like growth factors (IGF) are evolutionarily highly conserved proteins that play an important role in growth and metabolism, but also in neurogenesis through their role in neuronal stem-cell homeostasis (reviewed in Lee et al., 2016; Ali et al., 2018). In *Drosophila*, expression of *dFMR1* specifically in the insulin-producing cells (IPSs) or in the whole nervous system is able to rescue the defective free-running locomotor rhythmicity of *dFMR1* mutants, as well as short-term and long-term memory defects in olfactory learning tests (Monyak et al., 2016). Protein levels of the major insulin-like peptide DILP2 are elevated in the IPCs of *dFMR1* mutants, suggesting a post-transcriptional control, and markers of the insulin pathway are up-regulated. Through fine genetic manipulations aimed at reducing insulin signaling (IS), the authors were also able to obtain a rescue of the circadian rhythmicity of *dFMR1* flies. These data are consistent with the fact that *FMR1* is expressed in the IPSs of the mammalian pancreas and that insulin and IGFs have been involved in many neurological events, including synaptogenesis and progenitor cell growth (reviewed in Fernandez and Torres-Alemán, 2012). Metformin is a drug used to cure type 2 diabetes that acts as a sensitizer of IS signaling by increasing Phosphatase and TENsin homolog protein (PTEN) expression, AMP-activated protein Kinase (AMPK) activation and decreasing Target Of Rapamycin (TOR) signaling. Treatment of *dFMR1* mutants with metformin restores normal short-term courtship memory and normal olfactory long-term memory (Monyak et al., 2016). Using GAL80, the temperature-sensitive repressor of GAL4, Monyak et al. (2016) also tested the precise stage at which reduction of IS is necessary to rescue the different behavioral phenotypes of *dFMR1* mutants. While lowering IS during adulthood is sufficient to rescue learning and memory in the olfactory-based paradigm, reduction during the pupal stages is indispensable to rescue circadian behavior. This may mean that dFMRP is required at different developmental steps to temporally modulate IS. The weight of KO *Fmr1* mice is significantly increased compared to wild-type (Dölen et al., 2007) and some FXS patients are obese (Nowicki et al., 2007) and show elevated IS through the mTor pathway in blood and brain (Hoeffer et al., 2012), implying that mis-regulation of the insulin pathway is likely to be one cause of the disease and a promising target for therapy (reviewed in Castagnola et al., 2017).

## Conclusions

Individuals affected by FXS show a broad spectrum of clinical presentations, with a great variability of signs, symptoms and severity levels (Hagerman, 2002). Indeed, due to the high number of FMR1 targets, FXS can be considered as a multifactorial disorder. The possibility to study null *dFMR1* flies and mice brought significant advantages for understanding the functional roles of FMRP. These systems made it possible to decrypt several pathways responsible for phenotypes recalling human symptoms, such as cognitive and learning deficits. In comparison to the other animal models, *Drosophila* is less expensive and easier to maintain, lays many eggs that enable to perform genetic screens, has a shorter lifespan and generation time, a smaller entirely sequenced genome (Adams et al., 2000) and sophisticated genetic tools to study human diseases like the easiness to make transgenics or to carry out *in vivo* functional studies (reviewed in Wangler et al., 2015; Ugur et al., 2016; Chow and Reiter, 2017). The fly genome has many orthologs displaying similar roles to human disease genes (Fortini et al., 2000; Doronkin and Reiter, 2008) that can be potential targets for functional and therapeutic studies. *dFMR1* mutants exhibit defects that resemble those observed in human FXS patients, and flies and humans share many pathways that are altered in the disease and likely responsible for a specific set of phenotypes. Studies on FXS in *Drosophila* have set a paradigm to validate drug targets and gain a deeper insight into their molecular mechanisms for future research on FXS and other neurodevelopmental diseases. This is also because the dissection of the correlation between pathways and genotypes can be more easily realized in the fly than in any other model system.

## Author contributions

All authors listed have made a substantial, direct and intellectual contribution to the work, and approved it for publication.

### Conflict of interest statement

The authors declare that the research was conducted in the absence of any commercial or financial relationships that could be construed as a potential conflict of interest.
